# Reprogramming Rotavirus: Reverse Genetics-Driven Design of Viral Vector Platforms

**DOI:** 10.3390/v18060655

**Published:** 2026-06-09

**Authors:** Ke Li, Xiao Wei, Xuanze Ouyang, Xiafei Liu, Pengdi Chai, Yu Bai, Zhaojun Duan

**Affiliations:** 1 NHC Key Laboratory of Medical Virology and Viral Disease, National Key Laboratory of Intelligent Tracking and Forecasting for Infectious Diseases, National Institute for Viral Disease Control and Prevention, Chinese Center for Disease Control and Prevention, Beijing 102206, China; like990301@163.com (K.L.);; 2 School of Public Health, Gansu University of Traditional Chinese Medicine, Lanzhou 730000, China; 3 The First Clinical Medical Institute, Henan University of Chinese Medicine, Zhengzhou 450000, China; 4 CAS Key Laboratory of Pathogen Microbiology and Immunology, Institute of Microbiology, Chinese Academy of Sciences, Beijing 100101, China; 5 Center for Drug Evaluation, National Medical Products Administration, Beijing 100163, China

**Keywords:** rotavirus, reverse genetics, vaccine vector, oral mucosal vaccine

## Abstract

Rotaviruses remain the leading cause of severe dehydrating diarrhea and associated mortality in children under five years of age worldwide. The successful global rollout of live-attenuated oral rotavirus vaccines has dramatically reduced rotavirus gastroenteritis morbidity and mortality, unequivocally demonstrating their excellent safety profile and potent induction of mucosal immunity. These attributes highlight the substantial potential of rotaviruses as novel oral mucosal vaccine vectors. Recent breakthroughs in reverse genetics, particularly the establishment of a fully plasmid-based system in 2017, have enabled precise insertion and stable expression of foreign antigens at targeted genomic loci. This advance has opened a viable pathway for developing multivalent oral mucosal vaccines. This review traces the historical development of rotavirus reverse genetics and summarizes the latest progress in its application as a vaccine vector platform. We focus on key strategies for foreign gene insertion and immunological outcomes in animal models, while critically evaluating persistent challenges—virus rescue efficiency, genetic stability of inserts, and limitations of current animal models—and outlining the rational design framework for RV-based vectors with improved stability, expression efficiency, and immunogenicity.

## 1. Introduction

Rotaviruses (RVs), belonging to the family Reoviridae, are a genus of non-enveloped, double-stranded RNA (dsRNA) viruses. The viral particles, approximately 70–75 nm in diameter, exhibit a characteristic wheel-like morphology under electron microscopy. The viral genome consists of 11 dsRNA segments with a total length of approximately 18.5 kb, encoding six structural proteins (VP1-VP4, VP6, and VP7) and six non-structural proteins (NSP1-NSP6) [1]. RVs are the leading pathogens causing severe dehydrating diarrhea in infants and young children, and remain a primary cause of mortality among children under five years of age [2]. In recent years, improvements in safe drinking water, sanitation, and medical services, coupled with the widespread implementation of preventive and therapeutic interventions including rotavirus vaccines, have led to a decline in associated infection and mortality rates [3,4]. Nevertheless, RVs remain a major contributor to the global burden of diarrheal diseases, continuing to pose a severe threat to child health [2].

Given the absence of specific antiviral therapeutics against RV infection, vaccination represents the most effective strategy for prevention. To date, four live-attenuated oral RV vaccines (Rotarix, RotaTeq, Rotavac, and Rotasiil) have been prequalified by the World Health Organization (WHO), while three others (LLR, Rotavin-M1, and the Wuhan hexavalent human-bovine reassortant vaccine) are licensed exclusively at the national or regional levels [5,6]. These oral vaccines have significantly reduced the morbidity and mortality of rotavirus gastroenteritis in children, demonstrating robust immunogenicity, the distinct advantage of inducing intestinal mucosal immunity, and generally excellent safety profiles. Despite this strong safety record, the rare but recognized risk of intussusception must be carefully evaluated when developing any new RV-based vectors [7]. With the rapid advancement of reverse genetics methods, researchers are now able to achieve targeted insertion and stable expression of foreign antigens at specific loci within the RV genome. This provides a feasible approach for constructing oral vaccine vectors and recombinant tool strains for functional studies [8,9,10]. While systemic vectors like Adenovirus and MVA excel at inducing rapid systemic immunity [11,12], the distinct advantage of RV vectors lies in their needle-free oral delivery and natural intestinal tropism, which efficiently stimulate targeted mucosal immunity. Consequently, this also renders RV vectors more susceptible to pre-existing immunity and the harsh gastric environment [5]. Building upon the inherent advantages of live-attenuated RV vaccines in terms of safety and mucosal immunity, this technological breakthrough has propelled the research and application of RVs as oral vaccine vectors, with their immunogenicity having been preliminarily validated in animal models [13,14].

This review systematically summarizes the research progress on rotaviruses as vaccine vectors, focusing on aspects including RV etiology and immunology, the development of reverse genetics technologies, RV vectors, and the immunological evaluation of oral vaccines in animal models. We highlight representative achievements published in recent years and discuss the potential and challenges of this platform in vaccine development, aiming to provide theoretical and technical references for the application of the RV platform in the design of novel vaccines.

## 2. Biological Foundations of Rotavirus as a Vector Platform

### 2.1. Virion Architecture and Structural Organization

**Figure 1:**
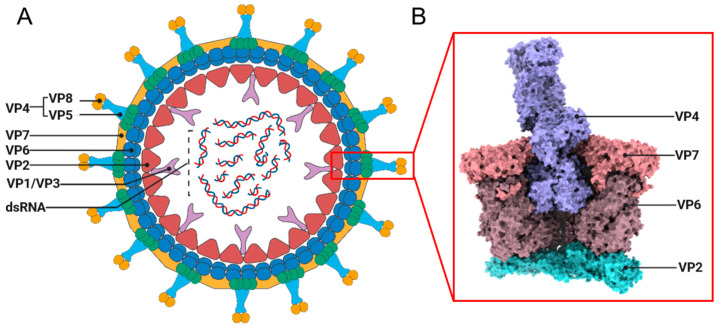
(**A**) Schematic Diagram of Rotavirus Structure. (**B**) Rotavirus structural protein schematic cross-section. PDB ID:8COA.

First isolated in animals and subsequently identified in human duodenal cells in 1973, RVs were formally named in 1978 based on their distinctive morphology [15]. Infectious rotavirus particles possess a characteristic triple-layered capsid structure (Figure 1A). The outer layer is composed of the VP7 glycoprotein and the spike protein VP4 (Figure 1B). Upon protease cleavage, VP4 yields the VP5* and VP8* subunits, which facilitate binding to host cell receptors. The intermediate layer consists of VP6, which serves as the primary basis for viral group classification (Figure 1B). The innermost core has an icosahedral structure primarily formed by the scaffold protein VP2 (Figure 1B), which encapsulates the viral genome, VP1, and VP3 [16]. Following the entry of infectious rotavirus particles into the host cell, the low calcium concentration within the endosome triggers the uncoating of the outer capsid, resulting in the formation of double-layered particles (DLPs) [17,18]. The release of these DLPs into the cytoplasm initiates rotavirus transcription [16].

### 2.2. Genome Organization and Functional Segmentation

The RV genome consists of 11 segments of dsRNA, ranging in length from 664 to 3302 base pairs (bp), with each open reading frame flanked by 5′ and 3′ untranslated regions (UTRs). All RNA segments possess a 5′ cap structure but lack a 3′ poly(A) tail. Segments 1–4 encode the structural proteins VP1-VP4, respectively, while segments 6 and 9 encode the structural proteins VP6 and VP7. Segment 5 encodes the non-structural protein NSP1; segments 7, 8, and 10 encode the non-structural proteins NSP3, NSP2, and NSP4, respectively; and segment 11 encodes the non-structural proteins NSP5 and NSP6.

The structural proteins VP1, VP2, and VP3 are located within the viral core and are essential for viral replication and transcription. VP1 acts as an RNA-dependent RNA polymerase (RdRp), responsible for the replication and transcription of viral RNA. VP3 exhibits guanylyltransferase activity and is involved in the capping of viral mRNAs, serving as a crucial enzyme during viral transcription. VP2 forms the major component of the viral core shell, providing a scaffold for the VP1 and VP3 enzymatic complex. The RV genome exhibits a high degree of genetic diversity. Based on variations in the VP6 gene sequence, RVs are classified into nine groups (A–D, F–J) [19]. Among them, species A, B, C, and H rotaviruses can infect humans. In contrast, the remaining species, including the recently identified K and L, predominantly infect animals [19,20]. VP4 and VP7 act as major neutralizing antigens. VP4 is cleaved into VP5* and VP8*, which mediate viral attachment and entry. Based on the genetic diversity of VP7 and VP4, group A rotaviruses can be further classified into at least 42 G (glycoprotein) and 58 P (protease-sensitive) genotypes [21].

Non-structural proteins such as NSP1 act as viral interferon (IFN) antagonists [22]. It inhibits the expression of type I IFN by antagonizing the functions of interferon regulatory factors (IRF3, IRF5, and IRF7) [23], thereby suppressing the host innate immune response. RV replication occurs in cytosolic inclusion bodies known as viroplasms, which are composed of viral and cellular proteins. NSP2 and NSP5 are essential for viroplasm biogenesis [24]. As a multifunctional enzyme, NSP2 also possesses nucleic acid helix-destabilizing, Mg2+-dependent nucleoside triphosphatase (NTPase), RNA triphosphatase (RTPase), and nucleoside diphosphate kinase (NDPK) activities [25]. NSP5 plays a role in multiple processes, including viroplasm dynamics and regulation. During viral genome replication, it acts as an adaptor linking NSP2 with various functional properties of other rotavirus proteins [26]. NSP6 is present within viroplasms and may indirectly regulate the phosphorylation state of NSP5 [25]. NSP3 facilitates the translation of rotavirus mRNAs and inhibits host cellular protein synthesis by antagonizing the poly(A)-binding protein (PABP) [27]. Furthermore, NSP4 not only functions as an intracellular receptor for newly synthesized DLPs but also acts as an endoplasmic reticulum (ER) transmembrane protein, mediating protein transport from the ER to the Golgi apparatus [28,29]. NSP4 is also characterized as a viral enterotoxin, capable of inducing diarrhea by modulating the phospholipase C-dependent calcium signaling pathway, which subsequently affects chloride secretion in intestinal villi [30].

### 2.3. Cell Entry, Replication Cycle, and Host Interactions

**Figure 2:**
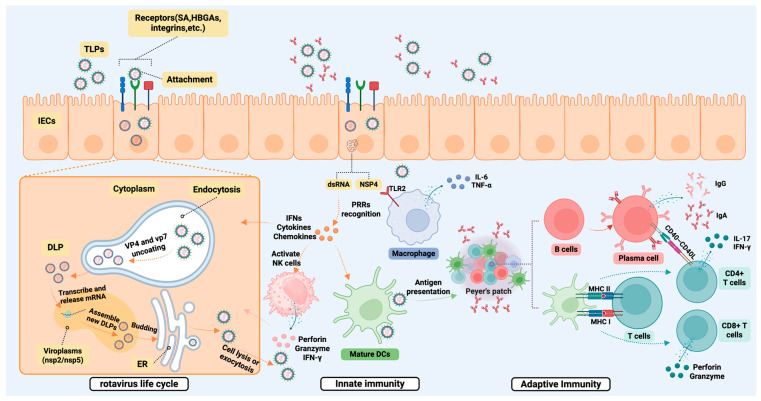
Host Immune Responses to Rotavirus Infection: From Viral Entry to Innate and Adaptive Immunity.

Infectious rotaviruses exist as triple-layered particles (TLPs). During viral entry, TLPs utilize the VP8* domain of the VP4 spike protein to recognize carbohydrate and protein receptors on the surface of intestinal epithelial cells (IECs). Subsequently, they enter the host cell via receptor-mediated endocytosis or direct penetration, and replicate within the cytoplasm [31]. Multiple receptors are involved in this process, primarily including sialic acid (SA), histo-blood group antigens (HBGAs), and integrins [32] (Figure 2). SA is one of the earliest identified major receptors. VP8* initiates attachment by binding to SA on the host cell surface, with a particularly strong dependence observed in neuraminidase-sensitive strains [33,34]. Although some viral strains can utilize internal sialic acid or other glycan receptors for binding, SA still plays a critical role in the entry process of most rotavirus strains [35]. In addition to SA, HBGAs are also essential carbohydrate receptors, serving as co-receptors for certain rotavirus strains. The binding of VP8* to HBGAs not only affects viral tissue tropism but also determines host susceptibility. For instance, certain P genotypes infect specific human populations through specific HBGA recognition [36,37]. Further studies indicate that integrins may participate in the viral entry process as transmembrane receptors, while the heat shock cognate protein 70 (Hsc70) interacts with the VP5* domain of VP4 during early attachment to facilitate viral entry into host cells [32,38].

Following receptor recognition, the virus enters the cell primarily via receptor-mediated endocytosis. Different strains may depend on either clathrin- or caveolin-mediated pathway. While direct membrane penetration was reported in a few early observations, subsequent experiments have confirmed that acidification-driven endocytosis is the universal mechanism [39]. After entering the endocytic vesicles, the low pH environment and fluctuations in calcium ion concentration trigger the rapid uncoating of the outer capsid (VP7 and VP4). VP5* undergoes a conformational rearrangement and inserts into the endosomal membrane to form a pore channel, ultimately delivering the DLPs into the cytoplasm [17]. In some instances, VP5* can directly disrupt the host cell membrane, allowing the virus to bypass endocytosis and enter the cytoplasm [40].

Upon entry into the cytoplasm, DLPs maintain their intact structure. The internal RNA-dependent RNA polymerase (RdRp) directly initiates transcription, synthesizing capped mRNAs and releasing them into the cytoplasm [41]. The newly synthesized mRNAs are immediately translated by host ribosomes into structural proteins (VP1–VP7) and non-structural proteins (NSP1–NSP6). Among these, the non-structural proteins NSP2 and NSP5 specifically interact to drive the formation of viroplasms. Viral genome replication, packaging, and the assembly of new DLPs are completed within these viroplasms [42] (Figure 2). The newly assembled DLPs subsequently bind to NSP4 on the ER membrane and enter the ER lumen via budding. During this process, they acquire a transient lipid envelope and the VP7 outer layer, maturing into TLPs bearing VP4 spikes. These TLPs are then transported along the microtubule network to the apical cell membrane via a dynein-mediated mechanism [43]. The mature viruses are ultimately released into the intestinal lumen through two pathways: cell lysis and a non-lytic vesicular transport or exocytosis mechanism (Figure 2). The latter allows for targeted apical release in polarized intestinal epithelial cells, thereby facilitating the infection of adjacent cells [44].

## 3. Immunological Basis for Rotavirus-Based Vaccine Design

Rotavirus infection triggers a multi-layered host immune response, primarily encompassing local intestinal mucosa-dominated innate defenses, as well as systemic adaptive responses centered on humoral and cellular immunity. These immunological layers act synergistically to accomplish viral clearance, prevent reinfection, and mediate vaccine-induced protection. Furthermore, they serve as critical immunological indicators for evaluating rotavirus vaccine efficacy.

### 3.1. Innate Immune Responses to Rotavirus Infection

Rotaviruses predominantly invade IECs (Figure 2). Following infection, viral double-stranded RNA (dsRNA) is recognized by intracellular pattern recognition receptors (PRRs), such as Toll-like receptor (TLR) 3, TLR7, TLR9, retinoic acid-inducible gene I (RIG-I), and melanoma differentiation-associated protein 5 (MDA5). This recognition activates downstream transcription factors IRF3 and NF-κB, inducing the expression of type I and type III interferons (IFN-λ), pro-inflammatory cytokines, and chemokines [45]. Furthermore, the non-structural protein NSP4, released during infection, acts as another pathogen-associated molecular pattern (PAMP). It is recognized by TLR2 on the surface of macrophages, further stimulating the secretion of cytokines such as Interleukin (IL)-6 and Tumor Necrosis Factor-α (TNF-α) [46]. Recent in vitro and ex vivo human blood studies reveal that a secreted glycoform of VP7 acts as an agonist for both TLR2 and TLR4 to activate innate immune cells [47]. The type I and type III IFNs secreted by IECs and these local immune cells rapidly establish a local antiviral state and promote the maturation of dendritic cells (DCs) [32,48]. Specifically, CD103+ DCs in Peyer’s patches and the lamina propria sample viral antigens and migrate to the mesenteric lymph nodes (MLNs) to present them to naive T cells, effectively bridging innate and adaptive mucosal immunity [49]. Concurrently, IL-15 produced by IECs via the TLR3/RIG-I/MDA5 pathways, alongside type I IFN and IL-15 secreted by plasmacytoid DCs, synergistically activates natural killer (NK) cells. Activated NK cells contribute to early preliminary viral control through perforin/granzyme-mediated cytotoxicity and additional IFN-γ secretion [50].

### 3.2. Humoral Immunity and Mucosal IgA Responses

Rotavirus infection significantly activates gut-associated lymphoid tissue (GALT), particularly B cells within Peyer’s patches. Mature DCs present viral structural proteins to naive T and B cells, thereby initiating B cell activation and differentiation into plasma cells that produce secretory immunoglobulin A (sIgA) [51,52] (Figure 2). RV-specific sIgA is the principal effector of long-term protection; it is initially detected in Peyer’s patches and subsequently expands to the intestinal lamina propria [53]. Elevated fecal sIgA levels are closely correlated with the viral clearance process, whereas IgA deficiency results in significantly delayed viral clearance and an increased risk of reinfection [54]. In addition to local mucosal responses, infection induces systemic humoral immunity, manifested by elevated serum IgA and IgG titers [55]. Systemic responses play a crucial role in restricting viral replication and alleviating clinical symptoms. Primary infection or vaccination predominantly generates specific neutralizing antibodies against homotypic serotypes, whereas reinfection or booster immunization can induce broadly reactive heterotypic neutralizing antibodies [56]. Serum IgA and IgG levels frequently correlate positively with clinical protection rates and are therefore widely utilized as indicators for evaluating vaccine immunogenicity [57]. In the GALT, IgA production relies on two parallel pathways: a T cell-dependent pathway, primarily driven by CD40-CD40L interactions and Transforming Growth Factor-β (TGF-β) signaling provided by CD4^+^ T cells; and a T cell-independent pathway, directly regulated by cytokines such as B-cell Activating Factor A Proliferation-Inducing Ligand (BAFF) and (APRIL) [58,59]. These two mechanisms collectively ensure the diversity and durability of the IgA response, providing a crucial theoretical basis for mucosal vaccine design.

### 3.3. Cellular Immunity and T Cell-Mediated Protection

Cellular immunity is indispensable for RV clearance and long-term protection. Mature DCs efficiently activate CD8^+^ T cells by cross-presenting viral structural protein antigens via MHC class I molecules. As the core effector cells for early viral clearance, activated CD8^+^ T cells directly target and lyse RV-infected IECs by releasing perforin and granzymes [60]. Simultaneously, DCs activate CD4^+^ T cells via MHC class II presentation. CD4^+^ T cells play a critical “orchestrating” role in the overall immune network: on one hand, they secrete IFN-γ and IL-17 to induce an antiviral state in target cells, recruit effector cells, and maintain the integrity of the intestinal mucosal barrier [61,62]. On the other hand, CD4^+^ T cells provide indispensable helper signals for B cell activation, isotype switching, and the production of sIgA through CD40-CD40L interactions and specific cytokine signaling, thereby laying the foundation for long-term mucosal immune memory [63]. Hosts lacking cDC1 exhibit not only delayed viral clearance and impaired CD8^+^ T cell responses but also compromised specific IgA responses, indicating that cellular immunity both directly participates in viral clearance and exerts synergistic effects by promoting humoral immunity, collectively fortifying intestinal mucosal and systemic immune defenses [64] (Figure 2).

## 4. Evolution of Rotavirus Reverse Genetics Systems

Reverse genetics (RG) is a technology that involves constructing complementary DNA (cDNA) of the full viral genome and expressing it in cells to ultimately assemble infectious viral particles de novo. Due to the complex structure of the RV genome and its replication dependency on specific viroplasm regions within the cytoplasm [65], the establishment of an RV reverse genetics system had faced immense challenges for a long time. Although a helper-virus-dependent RV reverse genetics system was established in 2006 [66], it was not until 2017 that Kanai et al. established the first entirely plasmid-based RV reverse genetics system [10], achieving a milestone breakthrough in RV reverse genetics technology.

### 4.1. Helper-Dependent Reverse Genetics Systems

In the early stages of RV RG development, researchers attempted to use the T7 RNA polymerase system combined with a helper virus to achieve gene segment replacement and recombinant virus rescue. In 2006, Komoto et al. [66] (Table 1) first reported the feasibility of this strategy: inserting the VP4-encoding cDNA into an expression vector containing a T7 promoter, and infecting COS-7 cells with the recombinant vaccinia virus (VV) strain rDIs-T7pol, expressing T7 RNA polymerase, for in vivo transcription. Subsequently, the human RV KU strain was infected as a helper virus, and its original VP4 expression was blocked using specific neutralizing antibodies. This preserved the recombinant VP4 generated by transfection, ultimately leading to the successful isolation of recombinant viruses carrying the heterologous VP4 in MA104 cells. Building upon this, Troupin et al. [67] constructed an NSP3 segment with a “tail repeat” structure, utilizing its preferential packaging characteristics during viral assembly to achieve the replacement of wild-type segments with rearranged segments. They reassorted the human RV NSP3 gene into the bovine RV RF strain and successfully rescued the recombinant virus after 18 consecutive passages. Despite the low efficiency, this study preliminarily validated the feasibility of using non-structural protein gene segments as foreign insertion sites. Furthermore, Trask et al. [68] established a temperature-sensitive (ts) mutation-assisted selection system, utilizing a ts VP4 mutant strain as the helper virus, and screening for recombinant viruses carrying a temperature-resistant mutant NSP2 segment under high-temperature conditions. Concurrently, siRNAs were utilized to inhibit the expression of the helper virus’s own NSP2, thereby significantly enhancing the selection pressure and improving the virus rescue efficiency. Although the aforementioned strategies were innovative in their technical approaches, they generally suffered from limitations such as complex operations, low efficiency, poor reproducibility, and applicability to only specific gene segments, making it difficult to meet the research demands for precise manipulation of the entire RV genome.

### 4.2. Evolution of Fully Plasmid-Based Reverse Genetics and Optimization Strategies

**Figure 3:**
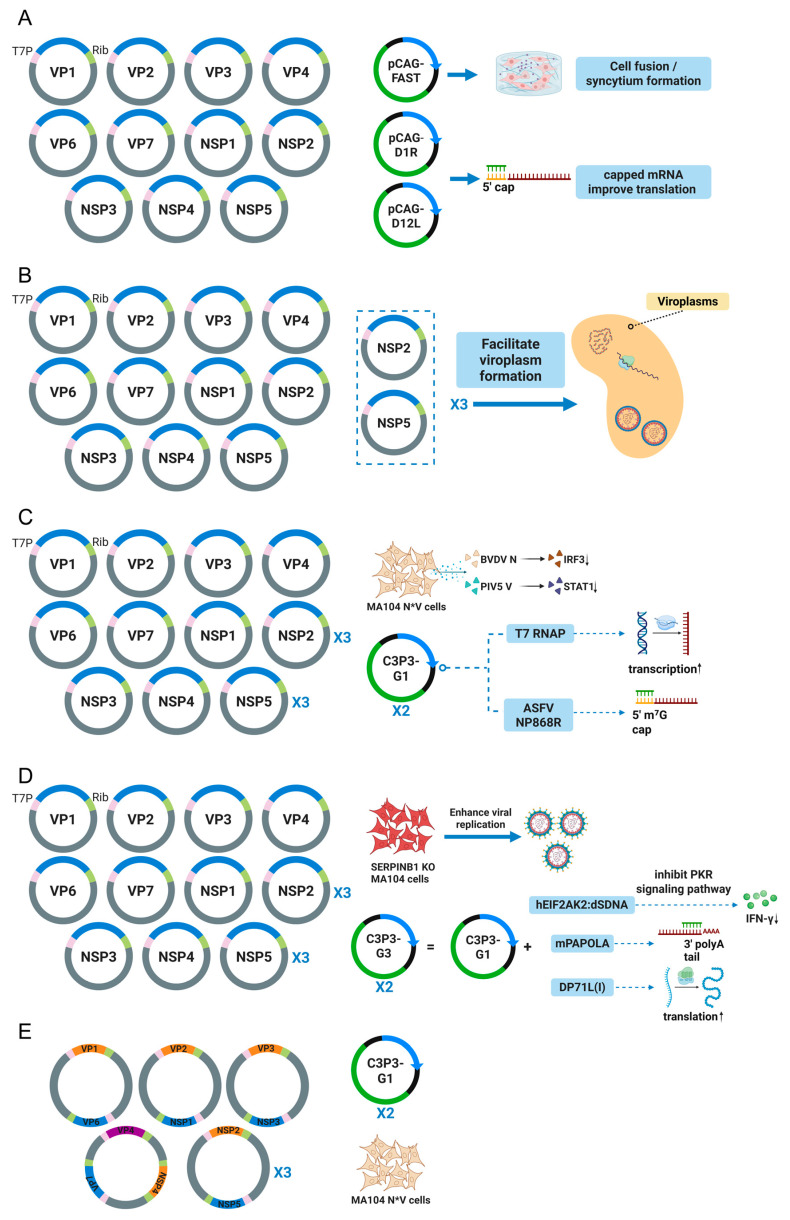
Evolutionary timeline of rotavirus reverse genetics systems and strategies for enhancing rescue efficiency. (**A**) 14-plasmid system: Comprises 11 RV genome plasmids and 3 helper plasmids. The FAST plasmid encodes a fusion-associated small transmembrane protein that induces syncytium formation (cell-to-cell fusion), creating a shared cytoplasmic pool that significantly increases the probability of colocalizing all 11 RV plasmids within a single functional unit. The D1R and D12L helper plasmids encode vaccinia virus capping enzymes to mediate the 5′ capping of T7-driven viral mRNAs, thereby stabilizing the RNA structures and enhancing translation. (**B**) 11-plasmid system: A completely helper-virus-free system where a 3-fold proportional increase in the NSP2 and NSP5 expression plasmids facilitates the optimal formation of viroplasms (intracellular sites for viral replication), robustly boosting rescue efficiency. (**C**) 12-plasmid system with C3P3-G1: Incorporates a 3-fold increase in NSP2/NSP5 and utilizes the C3P3-G1 plasmid, which expresses the T7 RNA polymerase and the ASFV capping enzyme NP868R. This streamlines the capping of T7 transcripts. The system is rescued in MA104-NV cells, which express viral antagonists to suppress host innate immune responses, maintaining a permissive environment for viral replication. (**D**) 12-plasmid system with C3P3-G3: Utilizes the upgraded C3P3-G3 plasmid. In addition to T7 polymerase and NP868R, it expresses DP71L to recruit protein phosphatase 1 (PP1), which dephosphorylates eIF2α and averts the translational shutoff triggered by PKR (hEIF2AK2) and dsDNA sensing. It also expresses mPAPOLA to polyadenylate and further stabilize viral transcripts. This system employs SERPINB1-knockout MA104 cells, effectively removing SERPINB1-mediated host restriction on rotavirus replication to maximize virus yield. (**E**) 5-plasmid system: A streamlined system incorporating a 3-fold increase in the combined NSP2/NSP5 expression cassette, utilizing the C3P3-G1 helper plasmid and MA104-NV cells to achieve high-efficiency rescue with minimized transfection complexity. Note: The arrows ↑ and ↓ indicate the enhancement and attenuation, respectively.

In 2017, Kanai et al. [10] established the first entirely plasmid-based RV reverse genetics system (Table 1, Figure 3A). Without the need for a helper virus infection, they developed a 14-plasmid system (11 plasmids from rotavirus + FAST + D1R + D12L) to successfully rescue the SA11 strain (Figure 3). The core of this system lies in co-transfecting the 11 plasmids encoding each RV gene segment along with three helper plasmids into BHK-T7 cells. The FAST protein enhances the replication efficiency of Reoviridae members that lack membrane fusion capability [66]. Concurrently, the co-transfection of plasmids encoding the vaccinia virus capping enzyme subunits D1R and D12L into T7 RNA polymerase-expressing cells facilitates the 5′ capping of nascent RNA transcripts, thereby stabilizing mRNA structures and robustly improving translation efficiency [73].

Despite this major breakthrough, the reliance on the FAST protein could induce early apoptosis and cytotoxicity, ultimately restricting viral rescue efficiency. To circumvent this limitation, Komoto et al. reported a highly optimized 11-plasmid system in 2018 (Table 1, Figure 3B). They eliminated the helper plasmids and increased the amount of NSP2 and NSP5 expression plasmids threefold [69]. Given that viroplasms—the essential cytoplasmic inclusion bodies for Reoviridae replication and initial virion assembly—are exclusively formed by NSP2 and NSP5 [74], the higher dosage of these plasmids significantly bolstered the viral recovery efficiency, resulting in a substantial increase in the rescued virus titers [69].

To further enhance rescue efficacy and broaden the system’s applicability to diverse RV strains, Sánchez-Tacuba et al. introduced a modified configuration in 2020 [70] (Table 1, Figure 3C). They incorporated C3P3-G1 into the 11-plasmid system, which simultaneously expresses the African swine fever virus NP868R capping enzyme and T7 RNA polymerase, thereby achieving efficient transcription and capping of viral RNA [75,76]. To create this cell line, researchers engineered MA104 cells to stably express the bovine viral diarrhea virus N protein and the parainfluenza virus 5 V protein. These proteins specifically targeted the degradation of IRF3 and STAT1—key factors in the interferon pathway [77,78], thereby robustly suppressing the host antiviral response. Utilizing this optimized platform, they successfully recovered refractory strains including human CDC-9, simian RRV, and murine D6/2-like [70]. Furthermore, Zhu et al. advanced this platform by pairing a next-generation helper plasmid, C3P3-G3, with a CRISPR/Cas9-screened SERPINB1-knockout MA104 cell line [71] (Table 1, Figure 3D). In addition to retaining the T7 RNA polymerase and the ASFV capping enzyme NP868R, C3P3-G3 introduces multiple functional elements that regulate translation and transcription processes. These include DP71L(I), which promotes eIF2α dephosphorylation; hEIF2AK2: dSDNA, which inhibits the PKR signaling pathway; and mPAPOLA, which enhances the 3′ poly(A) modification of mRNAs [79]. This comprehensive optimization drastically increased the recovery yield, exhibiting distinct advantages for rescuing low-titer strains or engineered constructs harboring large foreign insertions [71].

In 2025, the RV RG platform was further simplified utilizing the Lanzhou lamb rotavirus (LLR) strain [72] (Table 1, Figure 3E). LLR is a licensed live attenuated oral vaccine in China with a well-established safety profile, serving as a promising viral vector backbone [80,81]. Based on this strain, Liu et al. integrated the 11 RV genomic segments into a streamlined 5-plasmid system. In this optimized platform, the discrete plasmid co-expressing NSP2 and NSP5 was increased threefold while utilizing MA104-N*V cells, demonstrating that consolidating gene segments constitutes a highly effective strategy to maximize transfection efficiency and viral rescue [72]. Collectively, these iterative optimizations have not only streamlined the viral rescue process but also established a highly robust and versatile platform for rotavirus engineering and next-generation vaccine development.

## 5. Engineering Rotavirus as a Viral Vector

Viral vectors are engineered platforms that utilize viral genomes as tools to deliver foreign genes or therapeutic molecules into target cells or tissues. They have been widely applied in gene therapy, vaccine development, and antiviral research. A critical feature of rotavirus vectors is that specific gene segments can maintain self-replication capability even after the insertion of foreign genes, allowing them to achieve antigen presentation without losing replication competence and immunogenicity. Current insertion strategies primarily focus on the coding regions of non-structural proteins. Wei, J et al. has demonstrated that *NSP1*, *NSP3,* and *NSP5* can accommodate foreign segments while maintaining replication [82]. Given the crucial role of NSP5 in viroplasm formation, current applied research more frequently focuses on the *NSP1* and *NSP3* loci to balance replication safety and assembly risks. Generally, *NSP1* tends to possess a larger insertion capacity and genetic robustness. In contrast, *NSP3* relies on a well-established 2A bicistronic toolchain, exhibits higher expression levels, and is reusable across different genetic backgrounds, making it more widely utilized in antigen display and vaccine prototype validation.

### 5.1. NSP1 as a Flexible Platform for Foreign Gene Insertion

As a non-structural protein encoded by rotavirus, NSP1 inhibits the host interferon signaling pathway by promoting the degradation of IRF3, IRF7, and STAT1 [25,83]. Although it plays an important role in immune evasion, it is not essential for viral replication under cell culture conditions [84]. Truncated or optimized NSP1 can stably accommodate multiple small genes, providing an advantage for its use as an insertion platform. Kanai et al. first inserted reporter genes, such as *NanoLuc* (*Nluc*), at the C-terminus of a truncated NSP1 in the SA11 background, successfully rescuing a replication-competent recombinant strain and validating the feasibility of *NSP1* as an insertion site [10] (Table 2). Subsequently, they systematically evaluated the tolerance of *NSP1* to various reporter genes (*Nluc*, *ZsGreen*, *AsRed2*) [85] (Table 2). By adopting a configuration that retains the N-terminal 27 amino acids of *NSP1* fused with the foreign gene to balance expression and replication fitness, their results demonstrated that foreign genes could be expressed during early passages. Building upon this, Hatazawa and Fukuda et al. inserted *Nluc*, *EGFP*, and *mCherry* in tandem into the truncated *NSP1*, constructing a replication-competent multi-reporter recombinant virus (Table 2). This indicated the potential of the *NSP1* locus for the in-frame expression of multiple genes [86]. However, the genetic stability of foreign genes in rotaviruses remains a key challenge. Inserted genes may undergo deletion or mutation during serial passaging, compromising their expression. To address this, Kanai et al. significantly improved stability by performing codon optimization on the inserted segments, making their codon usage bias and GC content more closely resemble native rotavirus sequences [87] (Table 2). Furthermore, they constructed a recombinant SA11 strain expressing the HSV-2 *gD2* antigen for oral immunization in suckling and adult mice. The results showed that 8-week-old mice not only produced high levels of anti-RV antibodies but also induced IgG responses against gD2 [13] (Table 2). Although the expression level of *NSP1* inserts is relatively low, expression and genetic stability can be enhanced by retaining the N-terminal 27 amino acids of *NSP1* and applying virus-like codon optimization. Concurrently, attention should be paid to potential changes in in vivo fitness and immunophenotypes following the attenuation of NSP1’s function as an interferon antagonist [25].

### 5.2. NSP3-Based Bicistronic Expression Systems

NSP3 is a non-structural protein encoded by segment 7 of the rotavirus genome. Its primary function is to surrogate the host PABP and bind to eIF4G, thereby facilitating the translation of viral mRNAs [25]. In infected cells, NSP3 is expressed at moderate levels and exhibits high transcription efficiency, making it an ideal gene segment for constructing rotavirus reporter systems and studying viral biology [95]. Moreover, by utilizing the mature 2A bicistronic strategy, *NSP3* allows for the co-expression of foreign proteins without compromising its own function. Consequently, it has been widely applied in antigen display and vaccine design. Philip et al. was the first to use a 2A peptide to link various fluorescent protein genes to the C-terminus of the *NSP3* ORF in the SA11 strain (Table 2). The constructed recombinant viruses could simultaneously express NSP3 and the fluorescent proteins while maintaining stable replication [88]. In 2021, utilizing the reverse genetics system for the simian SA11 strain, the group further evaluated the feasibility of inserting different functional domains of the SARS-CoV-2 spike protein into the *NSP3* region [9] (Table 2). The results revealed that shorter segments (*RBD*, *ExRBD*) exhibited sustained expression stability for a defined number of passages (up to P5). In contrast, the full-length *S1* was highly susceptible to gene loss, yielding extremely low or undetectable expression. This suggests that the length of the foreign segment significantly impacts expression efficiency and genetic stability. To overcome this limitation, researchers further optimized the antigen design and constructed an rSA11/S1f expression platform capable of glycosylation modifications (Table 2). This recombinant strain stably expressed a glycosylated S1 protein of approximately 120 kDa, which could be recognized by anti-SARS-CoV-2 antibodies and bound to the ACE2 receptor [91].

In 2022, Diebold et al. inserted the *RBD* and *RBM* of SARS-CoV-2 into the C-terminus of *NSP3* in the background of the bovine RF strain (Table 2). Their results demonstrated that the *RBM* insert performed well in terms of replication efficiency, genetic stability, and induction of immune responses [8]. Building on this, Liu et al. further constructed the rLLR/NSP3-CoV2/RBD recombinant virus using the Lanzhou lamb rotavirus (LLR) strain (Table 2). This recombinant strain stably expressed the RBD protein up to passage 5 (P5) while retaining robust infection and replication capabilities [92]. In addition to SARS-CoV-2 antigens, the *NSP3* platform has been utilized to express the major capsid protein VP1 and its subdomains of human norovirus. In 2022, Philip et al. inserted the norovirus *VP1, P*, and *P2* domains into the *NSP3* region of SA11 (Table 2). The results showed that the P and P2 segments were stably expressed and formed conformationally correct dimers recognizable by conformation-dependent antibodies. Conversely, the larger *VP1* segment underwent deletion during serial passaging [89]. Furthermore, an Nluc-tagged murine recombinant rotavirus (rD6/2-2g) has been developed for real-time monitoring of in vitro and in vivo infections via bioluminescence imaging [90] (Table 2). Concurrently, the immunological applications of the *NSP3* insertion platform have been validated in animal models. Kawagishi et al. constructed a recombinant strain expressing the intact human norovirus VP1 based on the simian RRV strain and validated its immunogenicity in mice (Table 2). Oral immunization induced a robust humoral response, with antibodies generated in serum and feces effectively neutralizing norovirus [14]. Recent advances have further expanded the capacity of these vectors. Moving beyond viral targets, researchers engineered the LLR strain via NSP3 to express Clostridium perfringens alpha-toxin, conferring robust cross-kingdom protection in animal models [93] (Table 2). Furthermore, to overcome single-gene packaging limits, Cheng X et al. simultaneously inserted heterologous *VP7* genes (G4 and G5) into the *NSP1* and *NSP3* segments [94] (Table 2). This yielded a stable trivalent (G4/G5/G9) vaccine, demonstrating the platform’s capability for multi-site, multivalent engineering. Taken together, current studies indicate that the NSP3 locus is most robust when accommodating small-to-medium segments.

In summary, as insertion sites for foreign genes, both *NSP1* and *NSP3* can accommodate large inserts of up to 2.2 k bp. However, they exhibit distinct trade-offs in vector design. Recombinant rotaviruses with foreign genes inserted into the *NSP3* segment demonstrate a higher proliferation capacity than those utilizing *NSP1*; however, these *NSP3*-recombinants are highly prone to rapid sequence deletion during replication. Conversely, the *NSP1* insertion strategy suffers from a relatively lower proliferation capacity and a potential subtle impact on in vivo immunogenicity due to the loss of its immune evasion function. Crucially, recombinant rotaviruses frequently exhibit genetic instability and potential loss of foreign inserts during serial passage, regardless of the insertion site. Therefore, while *NSP1* can accommodate multi-gene tandem expression for multivalent vaccine design, maintaining the integrity of the foreign sequences requires careful monitoring and strict control of the passage number.

### 5.3. Alternative Engineering Strategies Beyond NSP1/NSP3

In addition to the *NSP1* and *NSP3* genes, foreign genes can also be inserted into the *NSP5* and *VP4* loci. In the SA11 vector, *GFP* was inserted at the C-terminus of *NSP5* via a 2A peptide sequence, which successfully maintained stable viral replication. Concurrently, the high expression efficiency of GFP enables its application in high-throughput neutralization assays [82]. Furthermore, Kotaki et al. performed a large-scale deletion of the SA11 *VP4* gene, retaining only 120 bp sequences at each of the 5′ and 3′ termini, thereby generating a single-round infectious virus. Utilizing this virus as a vector, SARS-CoV-2 *RBD* were successfully inserted, validating the potential of the VP4-deficient virus as a viral vector platform [96]. However, while its single-round replication ensures high safety, the lack of in vivo viral spread limits mucosal antigen exposure and may ultimately compromise overall immunogenicity. These strategies significantly expand the repertoire of insertion sites for foreign genes; nevertheless, they still face critical challenges, including insufficient expression stability and immunosuppression.

## 6. Key Challenges

Leveraging their natural intestinal tropism, RVs offer an ideal platform for oral multivalent vaccines, having successfully expressed antigens (e.g., SARS-CoV-2, HSV-2, norovirus) at the *NSP1/NSP3* loci. The concurrent induction of immune responses against both RV and the norovirus antigen provide a preliminary proof-of-concept for multivalent oral vaccines. Beyond vaccines, RV can also serve as an oral delivery vector for antibody fragments or immunomodulatory molecules. Despite these promising prospects, clinical translation still faces numerous challenges:

1. Genetic Stability of Inserted Long Sequences. Foreign fragments approaching or exceeding 1.5–2.0 kb are prone to deletion or expression attenuation during passaging. Stability and expression can be enhanced through virus-like codon and GC content optimization, truncation into stable domains/epitopes, strict preservation of 5′/3′ UTRs and terminal secondary structures, and the introduction of signal peptides and glycosylation sites for secreted proteins.
2. Enhancement of Immunogenicity. Expression levels directly restrict immunogenicity, and baseline differences between *NSP3/NSP1* can amplify or limit the effects. It is recommended to enhance antigen accessibility and expression, balance immunogenicity and stability during fragment selection, and synchronously fortify systemic and mucosal immunity through oral multi-dose homologous boosting or oral-intramuscular heterologous prime-boost regimens.
3. Animal Model Limitations. Suckling mice are convenient but have limited clinical relevance, and adult mice are naturally tolerant to RV. It is recommended to use gnotobiotic pigs as the core model for viral challenge and immunological evaluation. This can serve as a hard endpoint to determine “protective efficacy” and provide a benchmark for expanding this platform to other enteric pathogens.

## 7. Future Perspectives

Despite significant advances in rotavirus reverse genetics and vector engineering, current strategies for constructing recombinant RV vectors remain largely empirical. A key future direction lies in the transition from empirical optimization to rational design, guided by mechanistic understanding. A rational design framework for RV-based vectors should integrate multiple layers of constraints, including genome organization, RNA structural stability, replication dynamics, and host–virus interactions. In particular, the insertion of foreign sequences must preserve the delicate balance between viral fitness and transgene expression. Disruption of RNA secondary structures or segment-specific packaging signals may lead to genetic instability, especially during serial passages. Recent insights into RNA chaperone activity, particularly the role of NSP2 in facilitating RNA remodeling and genome packaging, suggest that local RNA structural flexibility is a critical determinant of replication efficiency. Therefore, optimizing not only codon usage but also RNA folding dynamics may significantly improve the stability of engineered segments. In addition, the incorporation of bicistronic expression strategies enables efficient co-expression of viral and heterologous proteins while minimizing genome disruption. However, the positioning and structural context of inserted sequences must be carefully tuned to avoid replication bottlenecks. Collectively, these considerations highlight the need for a systematic and predictive framework that integrates sequence design, structural modeling, and functional screening. Such an approach will accelerate the development of next-generation RV vectors with improved stability, expression efficiency, and immunogenicity.

## Figures and Tables

**Table 1:** Evolution of rotavirus reverse genetics technology.

Year & Author	System Type	Cell Line	Key Innovations
2006, Komoto et al. [66]	Helper virus-dependent system	COS-7 and MA104	First helper virus-dependent plasmid system.
2017, Kanai et al. [10]	14-plasmid system	BHK-T7 and MA104	First completely helper-virus-free system (11 RV + 3 helper plasmids: FAST, D1R, D12L).
2018, Komoto et al. [69]	11-plasmid system	BHK-T7 and MA104	Helper-plasmid-free system (achieved via 3-fold increase in NSP2/NSP5 plasmids).
2020, Sánchez-Tacuba et al. [70]	12-plasmid system	BHK-T7 and MA104 N*V cells	Enhanced rescue efficiency using C3P3-G1 and MA104 N*V cells.
2024, Zhu et al. [71]	12-plasmid system	BHK-T7 and MA104 SERPINB1 KO cells	Optimized rescue using C3P3-G3 and SERPINB1 KO MA104 cells.
2025, Liu et al. [72]	5-plasmid system	BHK-T7 and MA104 N*V cells	Streamlined 5-plasmid system (with 3-fold increase in combined NSP2/NSP5 plasmid).

**Table 2:** Overview of representative *NSP1/NSP3* exogenous gene insertion studies.

Year & Author	Strain	Insertion Site	Inserted Gene (bp)	Genetic Stability	Key Innovations
2017, Kanai et al. [10]	SA11	*NSP1* ORF	*NLuc* (516)	≥P5	First fully plasmid-based RG with reporter insertion
2019, Kanai et al. [85]	SA11	*NSP1* ORF	*NLuc* (516), *ZsGreen1* (753), *AsRed2* (678)	≥P10 (by NSP1 truncation)	Stable reporter system established
2020, Philip et al. [88]	SA11	*NSP3* C-terminus	*UnaG* (699), *mRuby* (678), *mKate* (711), *TagBFP* (~720)	≥P10	First use of NSP3-2A for heterologous protein expression
2021, Hatazawa & Fukuda et al. [86]	SA11	*NSP1* ORF	*NLuc* (516), *EGFP* (717), mCherry (711); three-gene cassette total 2160 bp	≥P10 (by NSP1 truncation)	First multi-gene expression in a single segment
2021, Philip et al. [9]	SA11	*NSP3* C-terminus	*RBD* (636), *ExRBD* (~900), *S2-CR* (~1100), *NTD* (~900), *S1* (2274)	RBD/ExRBD/S2-CR ≥ P10; NTD P3–P5; S1 P2	Demonstrated CoV-2 antigens; strong length constraint
2022, Philip et al. [89]	SA11	*NSP3* C-terminus	HuNoV *VP1* (1620), *P* (~1100), *P2* (~840)	P/P2 ≥ P5; VP1 deletion/rearrangement on passage	Expression of human norovirus antigens
2022, Zhu Y et al. [90]	rD6/2-2g	*NSP3* C-terminus	*NLuc* (516)	≥P8	Defined tissue tropism, replication dynamics, transmission
2022, Diebold et al. [8]	RF	*NSP3* C-terminus	*RBD* (636), *RBM* (204)	RBD partially unstable; RBM ≥ P10	Epitope-level antigens show long-term stability
2023, Kanai et al. [87]	SA11	*NSP1* ORF	*NLuc* (516), *EGFP* (717), *mCherry* (711), *AsRed2* (678)	Optimized ≥P10	Introduced virus-like codon optimization strategy
2023, Philip et al. [91]	SA11	*NSP3* C-terminus	SARS-CoV-2 *S1* (2274)	≥P5	First stable expression of glycosylated S1
2023, Kawagishi et al. [14]	RRV	*NSP3* C-terminus	HuNoV *VP1* (1620)	P3	Oral inoculation in infant mice induced NoV-neutralizing antibodies
2024, Kawamura et al. [13]	SA11	*NSP1* ORF	HSV-2 *gD2* (~1185)	-	First expression of HSV-2 antigen in RV
2024, Liu et al. [92]	LLR	*NSP3* C-terminus	SARS-CoV-2 *RBD* (636)	≥P5	First CoV-2 antigen expression on the LLR vaccine backbone
2026, Wang J et al. [93]	LLR	*NSP3* C-terminus	*CPA-CTD* (372)	≥P12	First bacterial antigen expression
2026, Cheng X et al. [94]	G9P [6]	*NSP1/3* C-terminus	*G4-VP7-CDS* (981), *G5-VP7-CDS* (981)	≥P7	Constructed multivalent vaccines via dual-site (*NSP1/NSP3*) insertion

## Data Availability

No new data were created or analyzed in this study. Data sharing is not applicable to this article.

## Abbreviations

The following abbreviations are used in this manuscript:

RVs	Rotaviruses
dsRNA	double-stranded RNA
DLPs	double-layered particles
UTRs	untranslated regions
RdRp	RNA-dependent RNA polymerase
IFN	interferon
IRF	interferon regulatory factors
NTPase	nucleoside triphosphatase
RTPase	RNA triphosphatase
NDPK	nucleoside diphosphate kinase
PABP	poly(A)-binding protein
ER	endoplasmic reticulum
TLPs	triple-layered particles
IECs	intestinal epithelial cells
SA	sialic acid
HBGAs	histo-blood group antigens
Hsc70	heat shock cognate protein 70
PRRs	pattern recognition receptors
TLR	Toll-like receptor
RIG-I	retinoic acid-inducible gene I
MDA5	melanoma differentiation-associated protein 5
PAMP	pathogen-associated molecular pattern
IL	Interleukin
TNF-α	Tumor Necrosis Factor-α
DCs	dendritic cells
NK	natural killer
GALT	gut-associated lymphoid tissue
sIgA	secretory immunoglobulin A
RG	Reverse genetics
ts	temperature-sensitive
Nluc	NanoLuc
LLR	Lanzhou lamb rotavirus
